# Genome sequence of *Streptomyces auratus* DSM 41897

**DOI:** 10.1128/mra.00506-25

**Published:** 2025-08-11

**Authors:** Braden Christenson, Imen Nouioui, Chris Daum, Marcel Huntemann, Rekha Seshadri, Craig Stephens

**Affiliations:** 1 Department of Chemistry and Biochemistry, Santa Clara University 7162, Santa Clara, California, USA; 2 Leibniz Institute DSMZ, German Collection of Microorganisms and Cell Cultures, Braunschweig, Germany; 3 DOE Joint Genome Institute, Lawrence Berkeley National Laboratory 1666, Berkeley, California, USA; 4 Department of Biology, Santa Clara University 7162, Santa Clara, California, USA; University of Maryland School of Medicine, Baltimore, Maryland, USA

**Keywords:** *Streptomyces auratus*, genome, antibiotics

## Abstract

We report the genomic DNA sequence of the actinomycete *Streptomyces auratus* DSM 41897. The genome assembled as two linear chromosomal contigs (4,434,406 bp and 3,992,024 bp) and a circular plasmid (12,008 bp), with an overall GC content of 71.2%, and 33 predicted biosynthetic gene clusters for potential secondary metabolites.

## ANNOUNCEMENT

The *Actinomycetota* phylum is a source of diverse bacteria known to produce highly sought-after secondary metabolites for clinical and industrial use (1). Within this group, *Streptomyces* have particularly been exploited for their production of antibiotics and other bioactive compounds (2, 3). The type strain *Streptomyces auratus* DSM 41897 was sequenced as part of the “Sequencing the genomes of 1,000 Actinobacteria strains” project at the U.S. Department of Energy (DOE) Joint Genome Institute to provide insight into the pharmacological potential of this species.

*S. auratus* DSM 41897 was provided by the Leibniz Institute DSMZ and cultured in Trypticase soy broth (DSMZ medium 535) at 28°C for 3 days with shaking at 120 rpm (4). Genomic DNA was extracted using the MasterPure gram-positive DNA Purification Kit (Biosearch Technologies). Genomic DNA was sheared to 10 kb using Covaris g-Tube. An SMRTbell template library was sequenced on a Pacific Biosciences (PacBio) Sequel II sequencer using 8M 1 SMRT cells and Version 1.0 sequencing chemistry with 1 × 900 sequencing movie run times (5). PacBio’s SMRT Link software performed quality control and adapter trimming under default conditions. Read error correction was performed by the Flye assembler (6). A total of 5,108,190 filtered subreads (read N_50_ = 5855 bases) were assembled using Flye v.2.8.1 under the following settings: –asm-coverage 100 –plasmid –genome-size 5000000 t 64 –pacbio-raw (fasta list). The resulting assembly totaled 8,438,454 bp (71.2% GC) in three contigs: a 4,434,406 bp linear chromosomal contig, a 3,992,024 bp linear chromosomal contig, and a 12,008 bp circular plasmid. Circularity of the plasmid assembly was confirmed with Circlator (7). The average coverage was 2,279×. CheckM2 was used to assess genome completeness (100%) and possible contamination (0.32%) (8).

The *S. auratus* DSM 41897 genome was annotated by the integrated microbial genomes genome annotation pipeline version 5.0.23 (9). A total of 7,454 genes were identified, including 7,300 protein-coding genes, 19 rRNA genes, and 71 tRNA genes. The sequence showed 98.6% average nucleotide identity (10) to the *S. auratus* AGR0001 draft genome sequence (GenBank accession no. CP072931) (11), but the DSM 41897 assembly is consolidated to three scaffolds, compared to 238 for AGR0001. Accurate prediction of biosynthetic gene clusters (BGCs) is facilitated by high-quality genome assemblies (12). BGCs in *S. auratus* DSM 41897 were identified using antiSMASH v.7.1.0 (13) with default software parameters. The genome contains 33 high-confidence BGCs (Table 1) (14), including nine with 80% or greater similarity to known clusters in the MIBig v.4.0 database (15). These BGCs may encode bioactive compounds with potential for biotechnological research applications.

**TABLE 1:** Biosynthetic gene cluster distribution of *S. auratus* DSM 41897

BGC class	Total	Products of most similar known cluster(s)
Non-ribosomally synthesized peptides	1	No prediction
Polyketides	9	iso-migrastatin/migrastatin/dorrigocin A/dorrigocinB/13-epi-dorrigocin A; naringenin; lysolipin I; phoslactomycin B; antipain
Ribosomally synthesized and post- translationally modified peptides	6	radamycin/globimycin; citrulassin D
Terpenes	3	No prediction
Other	10	legonoxamine A/desferrioxamine B/legonoxamine B, Ectoine
Hybrid	4	Ulleungdin

## Data Availability

The genome of *Streptomyces auratus* DSM 41897 is deposited in GenBank under accession number NZ_JBNGCB000000000, with the respective annotated DNA sequences available as JBNGCB010000001.1, JBNGCB010000002.1, and JBNGCB010000003.1. Raw data are available as SRA accession number SRX7194182.
